# Organic Cation Transporter-Mediated Accumulation of Quinolinium Salts in the LV Myocardium of Rodents

**DOI:** 10.1007/s11307-022-01728-y

**Published:** 2022-04-20

**Authors:** Hilbert Grievink, Ofer Shamni, Seweryn Krajewski, Łukasz Steczek, Dirk Gründemann, Eyal Mishani, Galith Abourbeh

**Affiliations:** 1 Cyclotron/Radiochemistry Unit, Hadassah Medical Organization and Faculty of Medicine, Hebrew University of Jerusalem, 91120 Jerusalem, Israel; 2 Synektik SA, Warsaw, Poland; 3 Department of Pharmacology, Faculty of Medicine and University Hospital Cologne, University of Cologne, Gleueler Straße 24, 50931 Cologne, Germany

**Keywords:** OCT, Quinolinium, Ammonium salts, PET, [^18^F]FEtQ, MPI, Organic cation transporter

## Abstract

**Purpose:**

Quaternary ammonium salts have demonstrated marked accumulation in the left ventricular (LV) myocardium of rodents and swine. To investigate the mechanism underlying this uptake, the present study examined the interaction of [^18^F]fluoroethylquinolinium ([^18^F]FEtQ) with the family of organic cation transporters (OCTs).

**Procedures:**

The cellular uptake of [^18^F]FEtQ into HEK293 cells, expressing human OCT1, -2, or -3 (HEK293-hOCT), and its inhibition by corticosterone was evaluated *in vitro*. The inhibitory effect of decynium 22 (D 22) *in vivo* was also studied, using PET/CT of HEK293-hOCT tumor-bearing mice. Furthermore, the distribution kinetics of [^18^F]FEtQ were determined in rats, with and without pre-administration of corticosterone, and following administration to a non-human primate (NHP).

**Results:**

The accumulation of [^18^F]FEtQ in HEK293-hOCT cells was 15–20-fold higher than in control cells and could be inhibited by corticosterone. *in vivo*, the uptake of [^18^F]FEtQ in the LV myocardium of corticosterone-treated rats was significantly reduced compared to that of untreated animals. Similarly, following administration of D 22 to HEK293-hOCT tumor-bearing mice, the peak tumor uptake of [^18^F]FEtQ was reduced by 40–45 % compared to baseline. Contrary to the distinct accumulation of [^18^F]FEtQ in the LV myocardium of rats, no cardiac uptake was observed following its administration to a NHP.

**Conclusions:**

The quinolinium salt derivative [^18^F]FEtQ interacts with the family of OCTs, and this interaction could account, at least in part, for the increased uptake in the LV myocardium of rodents. Nonetheless, its low affinity for hOCT3 and the results of PET/CT imaging in a NHP indicate a limited clinical applicability as a radiopharmaceutical for cardiac and/or OCT imaging.

**Supplementary Information:**

The online version contains supplementary material available at 10.1007/s11307-022-01728-y.

## Introduction

Organic cation transporters (OCTs) belong to the solute carrier (SLC) 22 family and are a class of plasma membrane transporters. Transport of organic cations by the three OCT subtypes OCT1 (*SLC22A1*), OCT2 (*SLC22A2*), and OCT3 (or extraneuronal monoamine transporter (EMT); *SLC22A3*) is electrogenic, bidirectional, Na^+^ and H^+^ independent, and similar among various species. OCTs mediate the physiological absorption and/or secretion of a broad range of structurally diverse organic cationic substances and are inhibited by various additional compounds that are not transported. Transported substrates of OCTs include endogenous compounds (*e.g.*, choline, dopamine, and norepinephrine), drugs (*e.g.*, cisplatin, metformin, and cimetidine), and various xenobiotics (*e.g.*, the neurotoxin: 1-methyl-4-phenylpyridinium (MPP) and the prototypic organic cation: tetraethylammonium (TEA)). Several cations (*e.g.*, decynium 22 (D 22)), neutral compounds (*e.g.*, corticosterone), and anions (*e.g.*, probenecid) inhibit OCTs, yet without being substrates of these transporters [1–3]. Although the interactions of the various OCTs with their substrates and inhibitors are not fully understood, broad overlaps between the substrate and/or inhibitor specificities, as well as tissue distribution patterns, distinct species, and subtype-specific differences exist [1–3].

In humans, OCT1 is most strongly expressed in the liver [1, 4], whereas in rodents it is strongly expressed in the liver and kidneys [1, 5, 6]. OCT2 is most strongly expressed in the kidney in both humans and rodents [3–6]. OCT3 shows a broader tissue distribution in both humans and rodents, with the strongest expression in the heart, skeletal muscle, liver, brain, and placenta [1, 4–6].

In addition to their expression in healthy tissues, OCTs have also been found to be differently expressed in tumors and a variety of cancer cell lines [7, 8]. *in vitro* upregulation or overexpression of OCT1, 2, and/or 3 was found to be associated with increased accumulation and cytotoxicity of platinum compounds (*e.g.*, cisplatin and oxaliplatin), whereas a reduction in OCT3 levels was found to underlie cisplatin resistance [7–10]. Furthermore, it was suggested that the potency of oxaliplatin-based chemotherapy against colorectal tumors could be associated with higher OCT2 expression in colorectal cancer [8]. In line with these findings, several studies have shown correlations between tumor OCT expression levels and anti-cancer treatment responses [11–14].

Recently, our group introduced a new a quinolinium salt-based positron emission tomography (PET) radiopharmaceutical, [^18^F]fluoroethylquinolinium ([^18^F]FEtQ), which was designed to increase the clinical applicability of previously studied carbon-11 labeled quaternary ammonium cations as potential PET myocardial perfusion imaging (MPI) agents [15]. In line with previous data, after its i.v. injection to rats, [^18^F]FEtQ displayed rapid renal clearance and increased accumulation in the heart, followed by the kidneys and the liver [15–18]. These initial investigations suggested [^18^F]FEtQ has potential as a PET-MPI probe [15]. The current study investigated whether [^18^F]FEtQ interacts with the three OCT isoforms, and whether this interaction could account for its accumulation in the left ventricular (LV) myocardium of rodents.

## Methods

### General

Triton X-100, poly-l-ornithine hydrobromide (0.1 mg/ml stock in 150 mM boric acid-NaOH pH 8.4), oxaliplatin (12.6 mM stock in water), cisplatin (2 mM stock in Dulbecco’s modified phosphate buffer saline (DPBS)), corticosterone (7.22 mM stock in 40 % propylene glycol: 60 % saline), and decynium 22 (D 22; 20 mM stock in DMSO) were purchased from Sigma-Aldrich, St Louis, MI, USA. Geneticin (G-419) and Dulbecco’s modified Eagle’s medium (DMEM) were purchased from Gibco, Paisley, UK. Fetal bovine serum, DBPS, and the Cell Proliferation Kit were purchased from Biological Industries, Rehovot, Israel. BCA Protein assay kit was obtained from Mercury, Israel. Corticosterone Enzyme Immunoassay Kit was purchased from Arbor Assays, Ann Arbor, MI, USA.

Sprague–Dawley (SD) rats (male, 9 to 11 weeks) and Hsd:Athymic Nude-Fox1nu mice (male, 4 to 5 weeks) were obtained from Envigo (Rehovot, Israel). Animals were allowed to acclimate for at least three days, prior to the imaging studies or inoculation of tumors, were routinely kept in 12-h light/dark cycles and provided with food and water ad libitum. All applicable institutional and/or national guidelines for the care and use of animals were followed.

One adult female monkey (*Macaca fascicularis*, weight 4.75 kg) was used in the present study. Animal care was in accordance with the Israeli National Institute of Health Guide for the Care and Use of Laboratory Animals. Studies were supervised and conducted under protocols approved by the Animal Research Ethics Committee of the Hebrew University of Jerusalem, and in accordance with its guidelines.

### Chemistry and Radiochemistry

The syntheses of FEtQ and [^18^F]FEtQ have been previously described [15]. The radiolabeling was performed by a fully automated one-step radiosynthesis using ethyltrifluoromethanesulfonate quinolinium as a precursor, in a 44-min process, including high-performance liquid chromatography (HPLC) purification. Overall, 9.5 ± 6.7 GBq was obtained (*n* = 19), with a mean radiochemical purity of 96.8 % ± 3.2 % and a mean radiochemical yield of 9.8 % ± 6.8 %, decay-corrected to the end of the bombardment.

### Cell Lines

HEK293 cells were stably transfected with the cDNAs of hOCT1, hOCT2, hOCT3, or the empty (pcDNA3) vector (EV), and were kindly provided by Prof. Gründemann, Department of Pharmacology, University Hospital Cologne, Germany [19, 20]. The cells were cultured at 37 °C in an atmosphere of 5 % CO_2_ and 95 % relative humidity, in DMEM (1 g/l glucose) with 300 μg/ml Geneticin. Specific hOCT1-3 expression in the various cell lines was verified by RT-PCR (see supplementary material).

### *In vitro [*^*18*^*F]FEtQ Uptake Assays*

HEK293 cells stably transfected with hOCT1, hOCT2, hOCT3, or EV were seeded and cultured in poly-l-ornithine pre-coated 6-well plates until they had reached ~ 75–90 % confluence. On the day of the experiment, the medium was replaced by pre-warmed (37 °C) Krebs–Ringer Henseleit buffer (125 mM NaCl, 25 mM HEPES pH 7.4, 5.6 mM ( +) glucose, 4.8 mM KCl, 1.2 mM KH_2_PO_4_, 1.2 mM CaCl_2_, 1.2 mM MgSO_4_) and plates were pre-incubated for 30 min at 37 °C. In inhibition experiments, the inhibitor (corticosterone) was added during the pre-incubation period. Cells were incubated with 0.37 MBq [^18^F]FEtQ for different time points (0–30 min), depending on the experiment. After incubation, the media were aspirated, and the cells were washed twice with ice-cold DPBS and solubilized with 1 % Triton X-100. The intracellular accumulation of radioactivity was determined using a gamma counter (2480 Wizard^2^ PerkinElmer, Germany). The total radioactivity taken up by the cells was normalized by the overall added radioactivity and by protein concentrations, as determined using a BCA protein assay kit.

### *In vitro [*^*18*^*F]FEtQ Binding Studies*

HEK293 cells stably transfected with hOCT3 and EV were seeded and cultured in poly-l-ornithine pre-coated 6-wells plates until they had reached ~ 75–90 % confluence. On the day of the experiment, the medium was replaced by pre-warmed (37 °C) Krebs–Ringer Henseleit buffer, and plates were pre-incubated for 30 min at 37 °C. Pre-warmed [^18^F]FEtQ was added to the wells at increasing concentrations (0–150 μM FEtQ), and the cells were incubated for 15 min at 37 °C. Subsequently, the solutions were aspirated, and the cells were washed twice with ice-cold DPBS and solubilized with 1 % Triton X-100. The intracellular accumulation of radioactivity was determined using a gamma counter. The total radioactivity taken up by the cells was normalized by the overall added radioactivity and by protein concentrations, as determined using a BCA Protein assay kit. Data analysis was performed using GraphPad Prism software, version 6 (GraphPad Software, Inc.).

### Xenografts

Athymic nude mice were anesthetized with isoflurane (1–2 % in oxygen) and injected subcutaneously (s.c.) in the right front flank with a suspension of 5 million HEK293 cells (in DMEM), which stably express hOCT1, -2, or -3. Tumors were allowed to develop for three weeks before PET/CT acquisitions.

### PET/CT Acquisitions of Rodents and Image Analyses

PET/CT acquisitions and image analyses were conducted as previously described [15, 21]. Animals were anesthetized with isoflurane (1.0–2.5 % in O_2_) and maintained normothermic using a heating pad. Following a CT attenuation-correction scan, PET acquisitions were carried out in list-mode using an Inveon™ MM PET/CT small animal-dedicated scanner (Siemens Medical Solutions, USA). Emission sinograms were normalized and corrected for attenuation, scatter, randoms, dead time, and decay. Image reconstruction was performed using Fourier rebinning and two-dimensional ordered-subsets expectation maximization (2D-OSEM), with a voxel size of 0.776 × 0.776 × 0.796 mm^3^. Image analysis and quantitation were performed using Inveon Research Workplace 4.2 (Siemens). Delineation of volumes of interest (VOIs) was performed by manual segmentation, based on the PET and CT images, and the corresponding time-activity curves (TACs) were calculated. The distribution of radioactivity was calculated as standardized uptake values (SUVs) normalized to the total body weight of the animal.

PET scans were started at the time of [^18^F]FEtQ injection via the lateral tail vein and lasted for 45–60 min, depending on the animal model. *in vivo* inhibition of OCT was carried out in SD rats following i.v. injection of corticosterone (3.3 mg/kg) in propylene glycol:saline (4:6), and in athymic mice following i.v. injection of decynium 22 (0.075 mg/kg) in cremophore EL:ethanol:saline (1:1:18). Inhibitor solutions were injected 5 min prior to injection of [^18^F]FEtQ. In the corticosterone studies, serum corticosterone levels were measured 3 min after injection, using a Corticosterone Enzyme Immunoassay Kit.

### PET/CT Acquisition of a Non-human Primate (NHP)

After an overnight starvation, anesthesia of the monkey was induced by intramuscular injection of Domitor (0.1 mg/kg) and ketamine (0.1 mg/kg), following which the animal was ventilated through an endotracheal tube, and volatile anesthesia was maintained using isoflurane (1.5–2.0 % in O_2_). Vascular access was prepared in the femoral vein for administration of [^18^F]FEtQ and saline, and the monkey was monitored continuously for body temperature, O_2_ and CO_2_ saturation, blood pressure, pulse, and breathing.

PET acquisition was carried out on a Discovery ST PET/CT (GE Healthcare, Milwaukee, WI, USA). The animal was placed in a supine position, and a CT transmission scan was carried out prior to the administration of [^18^F]FEtQ (94.2 MBq). A 15-min dynamic emission scan focusing on the thorax was started 10–15 s before [^18^F]FEtQ injection, followed by 3 additional whole-body static scans (10 min each). Scans were acquired in a two-dimensional model, normalized, and corrected for randoms, dead time, scatter, decay, and attenuation. Images were reconstructed using OSEM iterative reconstruction.

### Statistics

Statistical analysis was made using GraphPad Prism software, version 6 (GraphPad Software, Inc.). Unless otherwise stated, data is expressed as mean ± SEM. Comparisons of [^18^F]FEtQ uptake were made using Student’s *T*-test, unless otherwise stated. The level of significance was regularly set at *p* < 0.05.

## Results

### *In vitro Uptake Studies Using hOCT1-3 Overexpressing Cell Lines*

Expression of hOCT1, -2, or -3 in HEK293 cell lines was verified by cDNA amplification, using specific hOCT1-3 primer pairs. The results (Fig. S1, supplementary material) indicate that each of the stably transfected cell lines only expresses a single hOCT isoform. HEK293 cells transfected with an empty vector (EV) do not express hOCT1-3 and were employed as control.

**Fig. 1.**
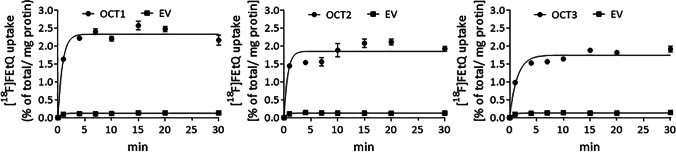
*In vitro* uptake of [^18^F]FEtQ in HEK293 cells stably transfected with human OCT1, -2, -3, or empty vector (EV). Each experiment was repeated twice using triplicate samples. Data are presented as mean ± SEM.

**Fig. 2.**
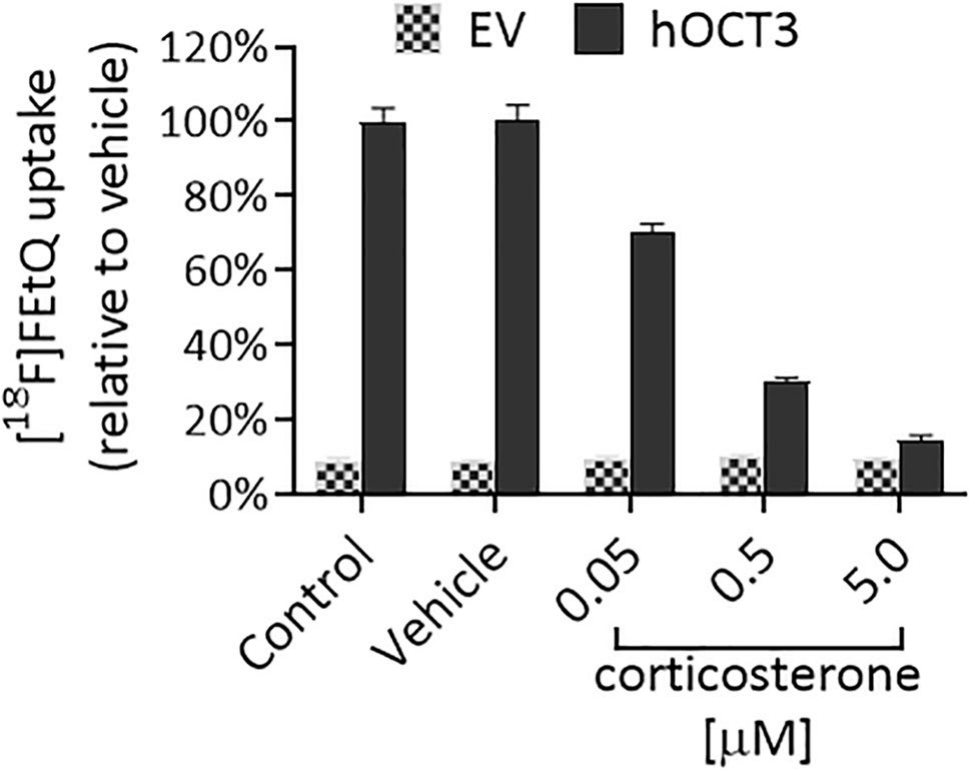
*In vitro* inhibition of [^18^F]FEtQ uptake (15-min incubation) by corticosterone in HEK293 cells stably transfected with hOCT3 or empty vector (EV). The experiment was repeated twice using triplicate samples. Data are presented as mean ± SEM.

OCT-mediated accumulation of [^18^F]FEtQ was investigated using the aforementioned four stably transfected HEK293 cell lines. The results shown in Fig. 1 reveal rapid uptake of [^18^F]FEtQ into hOCT1-3 expressing cells, which reaches a plateau after 5–10 min. The accumulation of [^18^F]FEtQ in these cells was 15–20-fold higher than in cells expressing the EV, in which essentially no accumulation of [^18^F]FEtQ was observed. Following incubation with corticosterone, a dose-dependent inhibition of [^18^F]FEtQ uptake into hOCT3-expressing cells was attained, with an IC_50_ of 0.05–0.5 μM (Fig. 2). These data indicate that [^18^F]FEtQ interacts with hOCT1, -2, and -3.

### *In vivo PET/CT Studies in Rats*

**Fig. 3.**
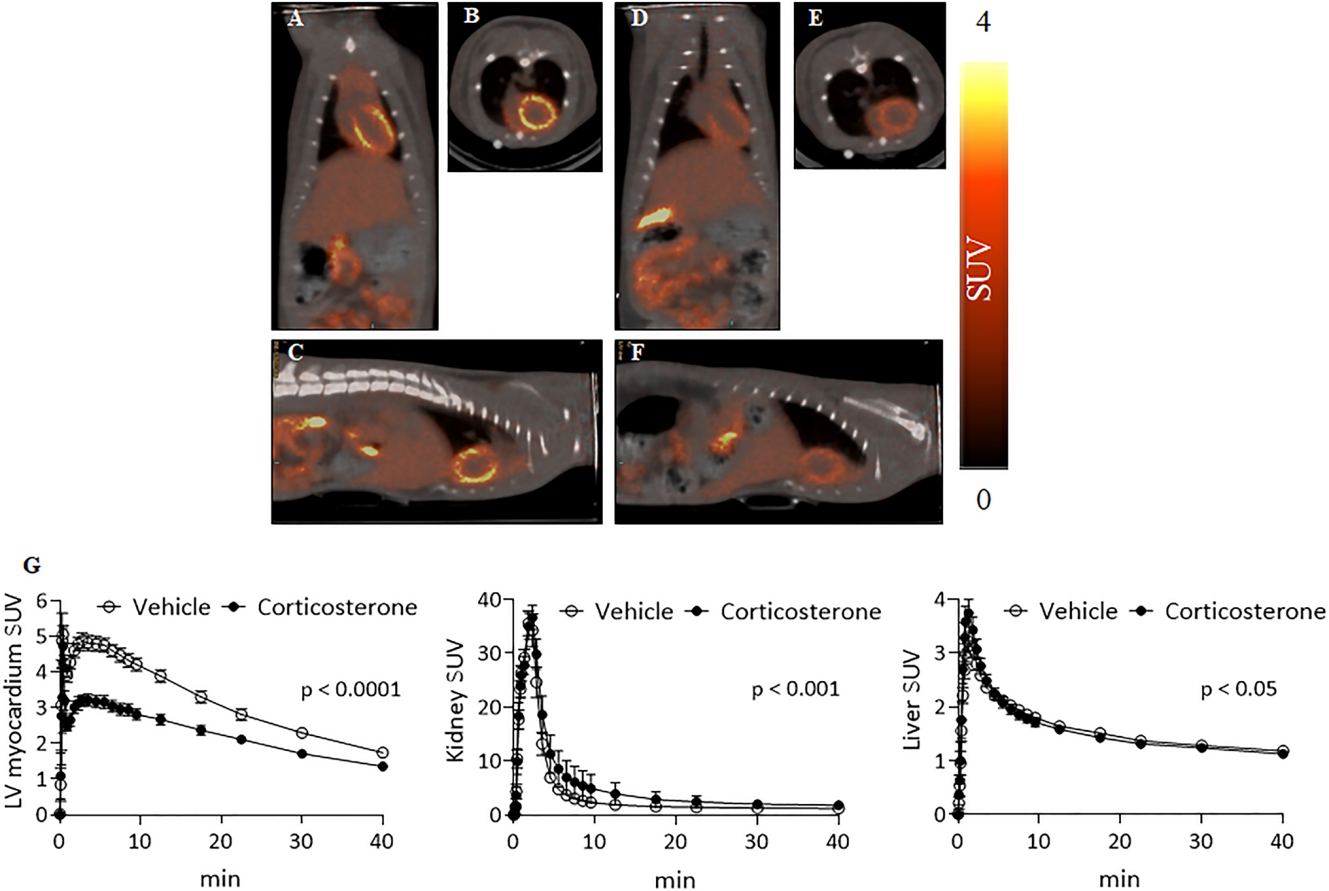
Representative PET/CT **a**, **d** coronal, **b**, **e** axial, and **c**, **f** sagittal slice images obtained following i.v. injection of [^18^F]FEtQ into male SD rats. Rats were pre-treated with either **a–c** vehicle or **d–f** corticosterone (3.3 mg/kg). Images represent the summation of 10–20-min frames. **g** Time activity curves of [^18^F]FEtQ uptake in the LV myocardium (left), kidneys (middle), and liver (right) of rats pre-treated with either vehicle or corticosterone. Data are presented as mean ± SEM (*n* = 7 per group). Comparisons were made using paired Student’s *t*-test.

Dynamic, 45-min PET acquisitions were carried out using adult male SD rats (310 ± 18 g, *n* = 14) starting at the time of [^18^F]FEtQ injection (15.8 ± 2.1 MBq, *n* = 14). In line with previous observations [15], [^18^F]FEtQ yielded good quality images, with rapid uptake and clear visualization of the LV myocardium, liver, and kidneys. Representative PET/CT images are shown in Fig. 3A–C. The major elimination route was renal, and the radioactivity uptake in the LV myocardium and liver was followed by pronounced washout from both organs (2.8- and 3.4-fold, respectively) throughout the 45-min scan (Fig. 3G).

Pre-treatment with corticosterone (3.3 mg/kg) had significantly reduced the accumulation of [^18^F]FEtQ in the LV myocardium (Fig. 3D–F) compared to vehicle injection (Fig. 3A–C), reaching peak SUVs of 3.2 and 4.9 at 3 min after administration of corticosterone *vs*. vehicle treatment, respectively (Fig. 3G). Furthermore, the renal clearance of [^18^F]FEtQ was delayed following corticosterone administration, resulting in a 40 % higher area under the kidney TAC compared to vehicle-treated rats. Albeit statistically significant, the difference between the liver TACs of the two treatment groups was less prominent. Serum corticosterone levels in blood samples taken from animals 3 min after corticosterone injection were 6.33 ± 1.66 μM, compared to 0.55 ± 0.16 μM in vehicle-treated rats.

### *In vivo Imaging of HEK-OCT Expressing Xenografts in Mice*

**Fig. 4.**
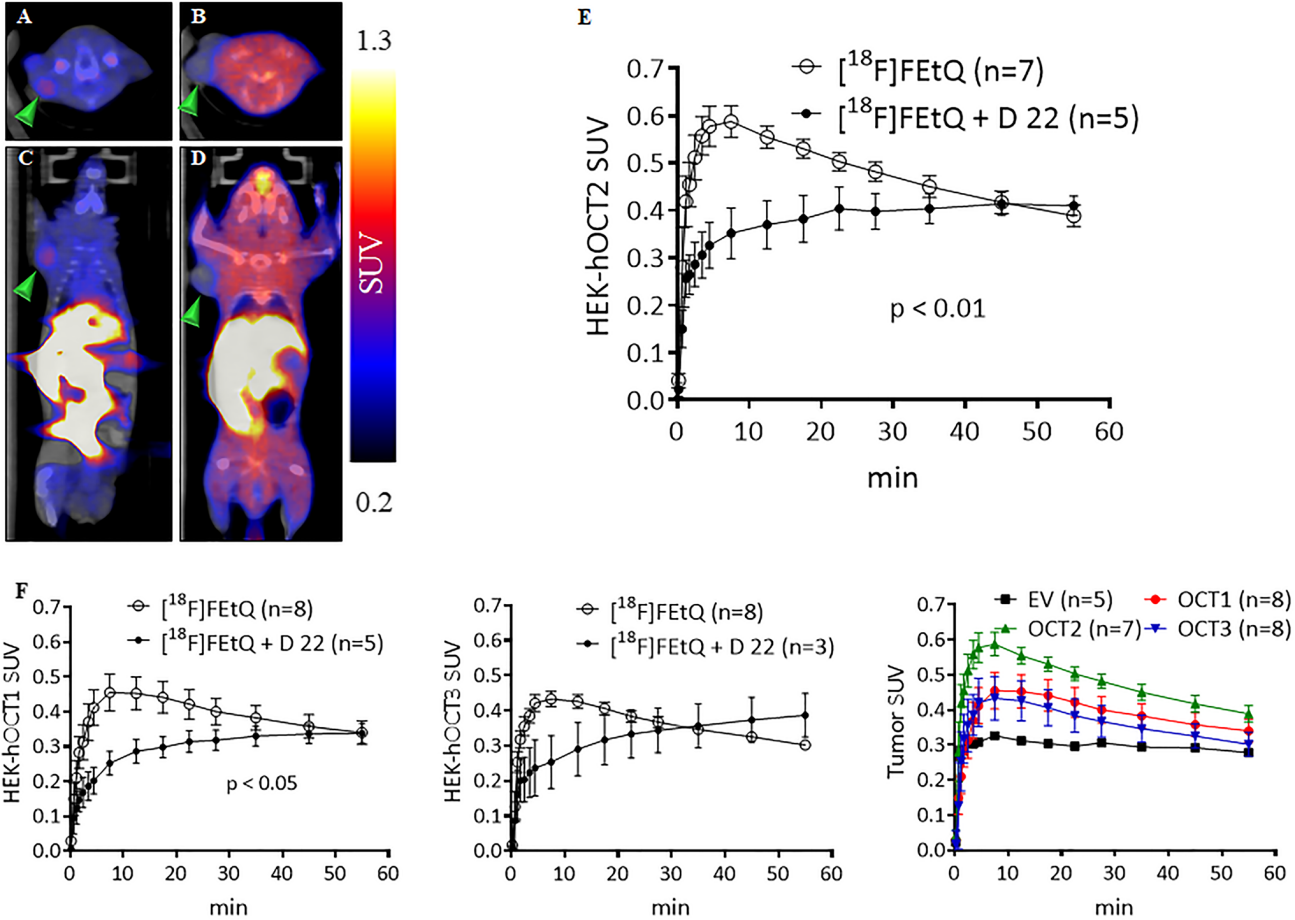
Representative **a**, **b** axial and **c**, **d** coronal PET/CT slice images (summation of 20–60-min frames) following i.v. injection of [^18^F]FEtQ into mice bearing HEK-hOCT2 tumors, **a**, **c** without and **b**, **d** with pre-administration of D 22 (0.075 mg/kg), and **e** their associated tumor TACs. **f** TACs representing [^18^F]FEtQ uptake in HEK-hOCT1and HEK-hOCT3 tumors are also shown. The combined graph shows [^18^F]FEtQ uptake in HEK-hOCT1, -2, or -3 expressing tumors, compared to control tumors expressing an empty vector (EV). Data are presented as mean ± SEM. Arrowheads point at the tumors.

Xenografts of HEK293 cells expressing hOCT1, -2, or -3 (HEK-hOCT1-3 tumors) or the empty vector (HEK-EV tumors) were established in athymic nude mice (30.8 ± 2.8, *n* = 28). Dynamic, 60-min PET acquisitions were started at the time of [^18^F]FEtQ injection (7.5 ± 0.9 MBq, *n* = 41) with or without pre-administration of D 22 (0.075 mg/kg). The results presented in Fig. 4A–E illustrate that the peak accumulation of [^18^F]FEtQ in HEK-hOCT2 tumors was 40 % lower in D 22-treated mice (SUV 0.35 *vs*. 0.59 at 8 min after injection). Moreover, while the TACs of HEK-hOCT2 tumors reveal rapid uptake of radioactivity followed by a moderate (33 %) washout over the remaining 50 min (Fig. 4E), pre-injection of D 22 abolished the apparent tumor washout, resulting in essentially identical tumor SUVs at 60 min after injection between D 22-treated and -untreated mice. Similar trends, albeit with less statistical significance, were observed in HEK-hOCT1 and HEK-hOCT3 tumors (Fig. 4F). All HEK-hOCT tumors exhibited significantly higher accumulation of [^18^F]FEtQ compared to tumors expressing the empty vector (*p* < 0.01; one-way ANOVA) (Fig. 4F).

### *In vitro Binding Studies*

**Fig. 5.**
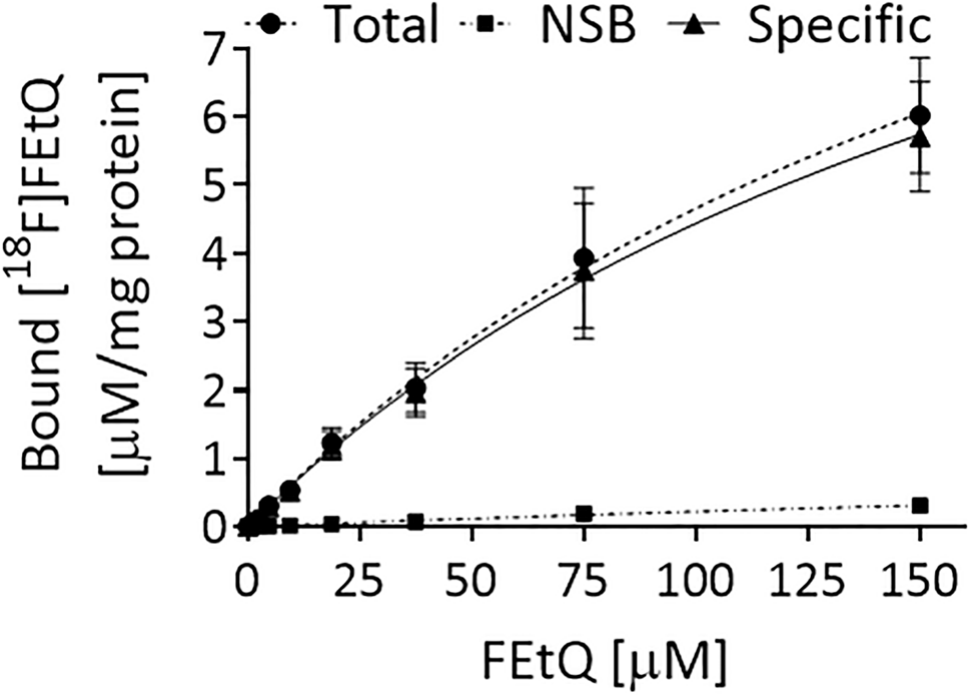
Specific binding of [^18^F]FEtQ in hOCT3-expressing HEK293 cells. Cells were incubated with increasing concentrations of [^18^F]FEtQ (0–150 μM, 37 °C, 15 min). Non-linear curve fitting yielded a *B*_max_ of 13.9 ± 4.9 μM/mg protein and a *K*_d_ of 213.5 ± 113.7 μM. Data are presented as mean ± SEM.

HEK293 cells stably expressing hOCT3 or empty vector (EV) were used for *in vitro* binding assays. Cells were incubated with [^18^F]FEtQ (0–150 μM) at 37 °C for 15 min. Human OCT3 expressing HEK293 cells were employed to determine the total binding (TB), whereas HEK293 cells expressing the EV were employed for the measurement of the non-specific binding (NSB). The specific binding (SB) was calculated by subtracting the NSB from the corresponding TB values at each FEtQ concentration. The [^18^F]FEtQ saturation binding curves, generated from two independent experiments (using triplicate samples), are shown in Fig. 5. Calculation of the specific binding parameters yielded a B_max_ of 13.90 ± 4.91 μM/mg protein and a K_d_ of 213.5 ± 113.7 μM.

### PET/CT of a NHP

**Fig. 6.**
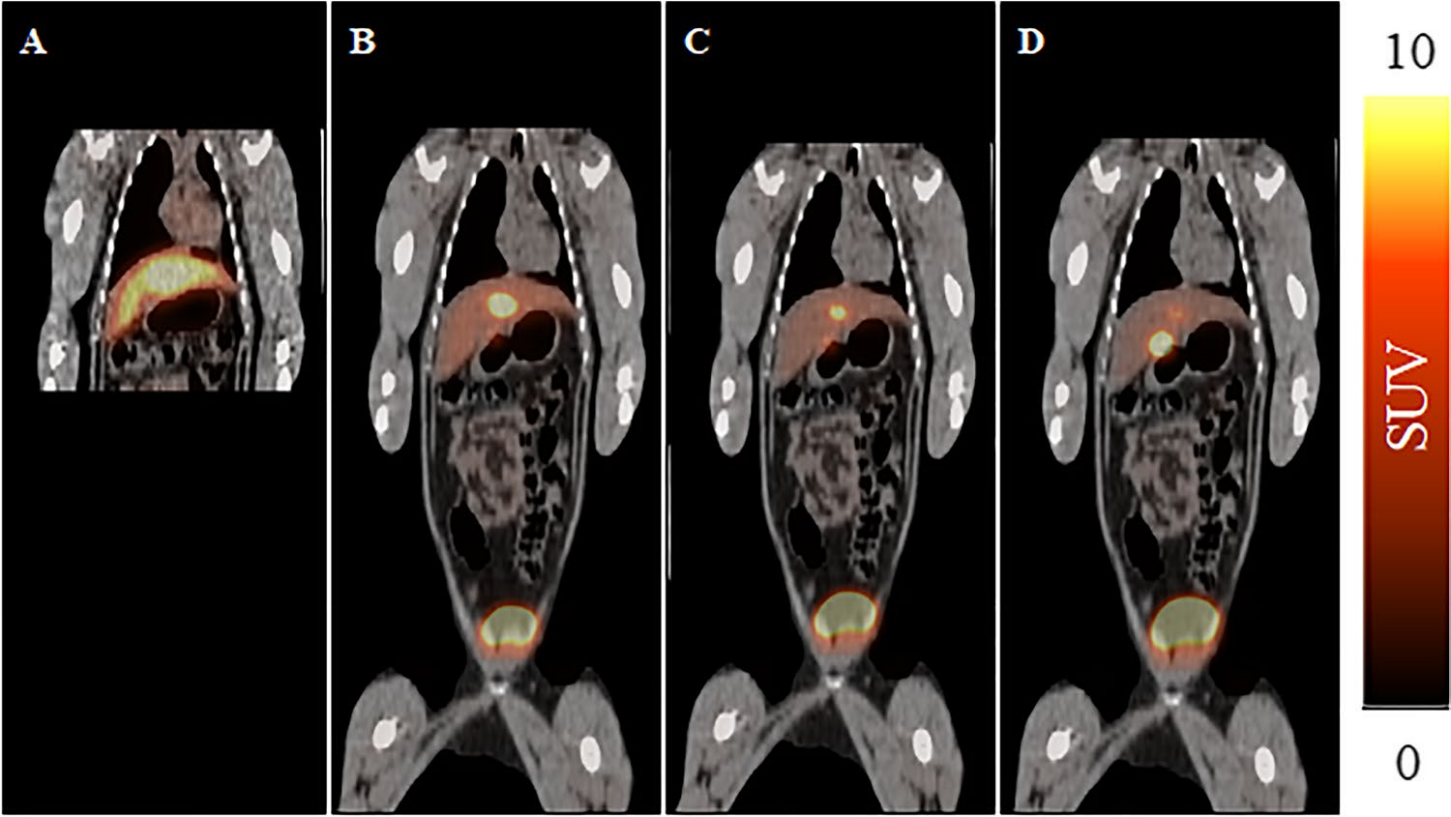
PET/CT coronal slice images following injection of [^18^F]FEtQ to a NHP. Images represent the summation of **a** 0–15-min, **b** 15–25-min, **c** 25–35-min, and **d** 35–45-min time frames.

The results presented in Fig. 6 illustrate that following injection of [^18^F]FEtQ to a monkey, no accumulation was observed in the LV myocardium throughout the 45-min acquisition. Similar to the results obtained in rodents, the major elimination routes were renal and hepatobiliary, resulting in the eventual accumulation of radioactivity in the gallbladder, intestines, and urinary bladder.

## Discussion

Over the past decade, our group has investigated the potential use of carbon-11 and fluorine-18-labeled quaternary ammonium salt derivatives for PET-MPI [15–18]. Studies investigating the uptake mechanism underlying the accumulation of quaternary ammonium salts in the LV myocardium suggested that it was not driven by mitochondrial or plasma membrane potentials, nor was it mediated via the Na^+^/K^+^ ATPase or the norepinephrine transporter (unpublished data). Taking into account the physicochemical properties and the distribution kinetics of the previously studied quaternary ammonium salt derivatives, a rapport with the family of OCTs has also been proposed, as their tissue distribution and substrate capacity align with the experimental observations [15–18]. The current study investigated whether [^18^F]FEtQ interacts with the OCTs, and whether this interaction is implicated in its accumulation in the LV myocardium of rodents.

*in vitro* uptake studies using HEK293 cells stably expressing hOCT1-3 revealed that [^18^F]FEtQ interacts with hOCT1-3 and that its accumulation in hOCT-expressing cells was 15–20-fold higher than in HEK293-EV cells (Fig. 1). Furthermore, the uptake of [^18^F]FEtQ into HEK293-hOCT3 cells was inhibited by corticosterone in a dose-dependent manner (Fig. 2) and at similar concentrations (IC_50_: 0.05–0.5 μM) to those reported in the literature (IC_50_: 0.3 μM) [1, 2]. Additional *in vitro* blocking studies targeting the hOCT1 and hOCT2 were not carried out owing to the lower potency of corticosterone for these isoforms (IC_50_: 22 μM and 34 μM, respectively) [1, 2, 22].

In rats, corticosterone is reported to be a more potent inhibitor of rOCT2 and rOCT3 (IC_50_: 4 μM and 5 μM, respectively), compared to rOCT1 (IC_50_: 150 μM) [1]. Of the three isoforms, rOCT3 has the strongest expression in the myocardium, whereas rOCT1 and rOCT2 expressions are predominant in the liver and kidneys, respectively [1, 4, 6]. Accordingly, following pre-treatment with corticosterone, the LV myocardium uptake and washout of [^18^F]FEtQ were significantly reduced, as illustrated by *in vivo* PET/CT studies (Fig. 3). Likewise, corticosterone administration had significantly delayed the renal excretion of radioactivity, yet its effect on the distribution kinetics in the liver was less conspicuous. These observations are in line with the fact that OCTs operate bidirectionally [1], and their inhibition is therefore expected to affect both transports into and out of the cells. Moreover, the measured serum corticosterone levels of pre-treated rats (6.33 ± 1.66 μM) were in good agreement with the observed inhibitory effects in the LV myocardium and the kidneys, and the reported IC_50_ values of corticosterone for rOCT2 and rOCT3 (4 μM and 5 μM, respectively). Owing to the lower potency of corticosterone towards rOCT1 (IC_50_: 150 μM), the distribution kinetics of [^18^F]FEtQ in the liver of corticosterone-treated rats were similar to those of vehicle-treated animals, albeit the difference was statistically significant (Fig. 3).

Decynium 22 is a cationic quinolinium derivative sharing a chemical structure motive with [^18^F]FEtQ (Fig. S2). It is one of the most potent inhibitors of hOCTs, having an approximately tenfold higher affinity towards hOCT3 (IC_50_: 0.09 μM), compared to hOCT1 and hOCT2 (IC_50_: 0.98 μM and 1.13 μM, respectively) [1, 2]. *in vivo* PET/CT studies of mice bearing xenografts of HEK293 cells expressing hOCT1, -2, or -3 revealed [^18^F]FEtQ accumulation in the various tumors, which was inhibited by D 22 (Fig. 4). These results indicate a specific interaction between [^18^F]FEtQ and hOCT1-3 *in vivo*. The observed high background activity in D 22-treated mice (Fig. 4B, D) emanates from the elevated blood activity following inhibition of OCT-mediated renal clearance of [^18^F]FETQ. The peak tumor uptake levels of all three HEK293-hOCT tumors were relatively low (SUV: 0.43–0.59) and varied between the different tumors. These observations could be explained by differences in hOCT1-3 expression levels and/or by different affinities of [^18^F]FEtQ for each isoform. Taking into account the reported potency of D 22 for hOCT3 (IC_50_: 0.09 μM) and considering its structural similarity to [^18^F]FEtQ, the latter was expected to result in better visualization of hOCT3-expressing xenografts. However, *in vitro* binding studies revealed a poor affinity of [^18^F]FEtQ for hOCT3 (K_d_ of 213.5 ± 113.7 μM) (Fig. 5), possibly accounting for the moderate accumulation of [^18^F]FEtQ in HEK293-hOCT3 xenografts. Nowadays, OCTs are recognized as low-affinity, high-capacity transporters [1]. Therefore, the poor affinity of [^18^F]FEtQ for hOCT3 is not atypical for OCT ligands and could explain the relatively low uptake of [^18^F]FEtQ in the hOCT1-3 expressing xenografts. Nonetheless, since injection of [^18^F]FEtQ to rats generated good quality images with clear visualization of the LV myocardium, it is plausible that the affinity of [^18^F]FEtQ for rOCT3 is higher compared to hOCT3. Further experiments to elucidate this hypothesis were beyond the scope of the current study.

To investigate whether the observed accumulation of [^18^F]FEtQ in the LV myocardium of rodents could be recapitulated in NHPs, a single study was carried out following i.v. administration of the radiopharmaceutical to a monkey. As opposed to the distinct accumulation of [^18^F]FEtQ in the LV myocardium of rats, no apparent uptake of the ligand was detected in the heart of the NHP (Fig. 6). A similar observation was reported by Carr et al. in the late 1970s, showing that radiolabeled analogues of the quaternary ammonium bretylium accumulated in the LV myocardium of rats, dogs, and pigs, yet not in that of monkeys or humans [23]. The extraneuronal uptake mechanism (uptake_2_) accountable for the cardiac accumulation of bretylium and its analogues was later identified to involve OCT3 [20, 24]. The discrepancies in the extent of [^18^F]FEtQ cardiac uptake between rats and the NHP could be attributed to differences in the extent of myocardial OCT3 expression between the two species and/or to different affinities of [^18^F]FEtQ for the respective OCT3 isoforms.

[^18^F]FEtQ was found to interact with OCTs, and this interaction is responsible, at least partially, for the accumulation in the LV myocardium of rodents. However, the present imaging in monkey indicates that this observation cannot be duplicated to NHPs, in line with previous reports about bretylium analogues [23]. The apparent low affinity of [^18^F]FEtQ for the hOCT3, and likely also to hOCT1 and -2, will limit its potential as a PET pharmaceutical for either MPI or OCT tumor screening.

## Conclusion

The quinolinium salt derivative [^18^F]FEtQ interacts with the family of OCTs, and this interaction could account, at least in part, for the increased uptake in the LV myocardium of rodents. Nonetheless, its apparent low affinity for hOCT3 and the results of PET/CT imaging in a NHP indicate a limited clinical applicability as a radiopharmaceutical for cardiac and/or OCT imaging.

## Supplementary Information

Below is the link to the electronic supplementary material.

Supplementary file1 (DOCX 34 KB)

Supplementary Fig. 1 (PNG 211 KB)

Fig. S1 A 2% agarose gel was loaded with PCR products generated from cDNA amplifications from HEK 293 cells transfected with the empty vector (EV; lanes: 1-4), hOCT1 (lanes: 5 & 6), hOCT2 (lanes: 7 & 8) or hOCT3 (lanes: 9 & 10). Lanes 1 & 5 were amplified with the hOCT1 (200 bp) primer pair. Lanes 2 & 7 were amplified with the hOCT2 (199 bp) primer pair. Lanes 3 & 9 were amplified with the hOCT3 (473 bp) primer pair. Lanes 4, 6, 8 & 10 were amplified with the 𝛽-actin (357 bp) primer pair. L= 100 bp ladder (Bio-Rad, Hercules, CA, USA). - = unloaded lane. As control for the intactness of mRNA, glyceraldehyde-3-phosphate dehydrogenase (GAPDH) mRNA was detected with specific primers. In separate experiments it was verified that the primers used to detect rOCT1 and rOCT2 do not show any cross-reactivity [26]. (TIF 46 KB)

Supplementary Fig. 2 (PNG 39 KB)

Fig. S2 Chemical structure of [18F]FEtQ and D 22 (TIF 103 KB)
