# Supplementary material for: Organic Cation Transporter-Mediated Accumulation of Quinolinium Salts in the LV Myocardium of Rodents
Source: Mol Imaging Biol. 2022 Apr 20;24(5):1–9. doi: 10.1007/s11307-022-01728-y (PMC9581852; doi:10.1007/s11307-022-01728-y)
Supplement: Supplementary file 1 — Supplementary file1 (DOCX 34 KB) [file 11307_2022_1728_MOESM1_ESM.docx]

**Supplementary material**

*RNA extraction and reverse transcriptase polymerase chain reaction (RT-PCR)*

Expression of the hOCT1, -2 or -3 was verified by RT-PCR. For this, total RNA was extracted from the various cell lines with TRI Reagent (Sigma-Aldrich, St Louis, MI, USA), according to the manufacturer’s instructions. RNA (1 μg) was reverse transcribed with M-MLV RT. cDNA was subsequently amplified using the GoTaq G2 Colourless Master Mix. The following primers were used to amplify the various DNA fragments [[2](#_ENREF_2), [25](#_ENREF_25)]: hOCT1-fw (*SLC22A1,* NM_003057.2; 200 bp): 5’-TCCCTCGAAGAGGATGTCA-3’, hOCT1-rv: 5’-ATTTCGACCAGAGCGGAGTA-3’; hOCT2-fw (*SLC22A2,* X98333.1; 199 bp): 5’-CGGAGATATCGGAGAACAGT-3’, hOCT2-rv 5’-GCATTCTTATTCTGGGAGATC-3’; hOCT3-fw (*SLC22A3,* AJ0001417.1; 473 bp): 5’-GTTTCGCTCTGTTCAGGTCTGTGT-3’, hOCT3-rv: 5’-TTATGTGTTCCCAGAAACTTC-3’; ß-actin-fw (*ACTB,* NM_001101.3; 357 bp): 5’-CACTCTTCCAGCCTTCCTTCCTG-3’, β-actin-rv: 5’-TAGTCCGCCTAGAAGCATTTGCG-3’. The following cycling protocol was employed for amplification of hOCT2, hOCT3 and β-actin: 95ºC for 2 min, 95ºC for 30 sec, 54ºC for 30 sec, 72ºC for 30 sec for 35 cycles, and a final extension at 72ºC for 5 min. The amplification of hOCT1 was conducted by employing the following cycling protocol: 95ºC for 2 min, 95ºC for 30 sec, 58ºC for 30 sec, 72ºC for 30 sec for 40 cycles, and a final extension at 72ºC for 5 min.
