# Supplementary figures and images for: Organic Cation Transporter-Mediated Accumulation of Quinolinium Salts in the LV Myocardium of Rodents
Source: Mol Imaging Biol. 2022 Apr 20;24(5):1–9. doi: 10.1007/s11307-022-01728-y (PMC9581852; doi:10.1007/s11307-022-01728-y)

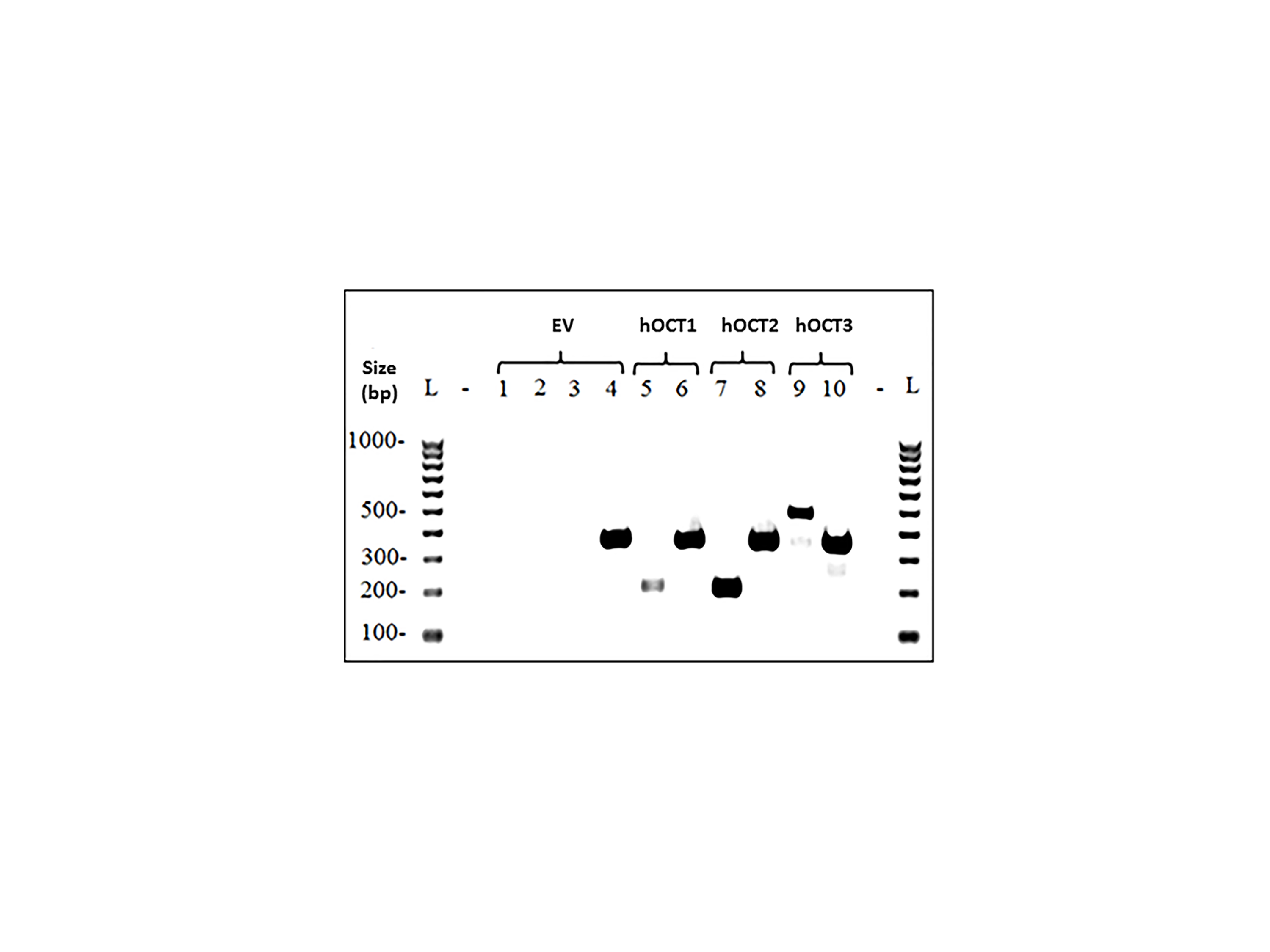

Supplement: Supplementary file 2 — (PNG 211 KB) [file 11307_2022_1728_Fig7_ESM.png]

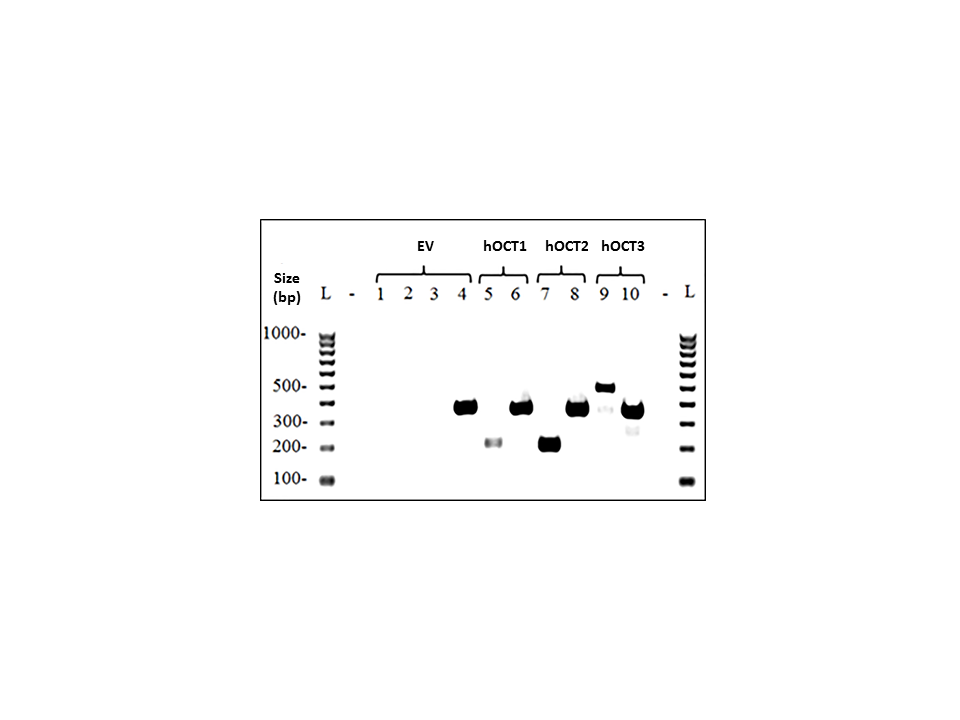

Supplement: Supplementary file 3 — Fig. S1 A 2% agarose gel was loaded with PCR products generated from cDNA amplifications from HEK 293 cells transfected with the empty vector (EV; lanes: 1-4), hOCT1 (lanes: 5 & 6), hOCT2 (lanes: 7 & 8) or hOCT3 (lanes: 9 & 10). Lanes 1 & 5 were amplified with the hOCT1 (200 bp) primer pair. Lanes 2 & 7 were amplified with the hOCT2 (199 bp) primer pair. Lanes 3 & 9 were amplified with the hOCT3 (473 bp) primer pair. Lanes 4, 6, 8 & 10 were amplified with the 𝛽-actin (357 bp) primer pair. L= 100 bp ladder (Bio-Rad, Hercules, CA, USA). - = unloaded lane. As control for the intactness of mRNA, glyceraldehyde-3-phosphate dehydrogenase (GAPDH) mRNA was detected with specific primers. In separate experiments it was verified that the primers used to detect rOCT1 and rOCT2 do not show any cross-reactivity [26]. (TIF 46 KB) [file 11307_2022_1728_MOESM2_ESM.tif]

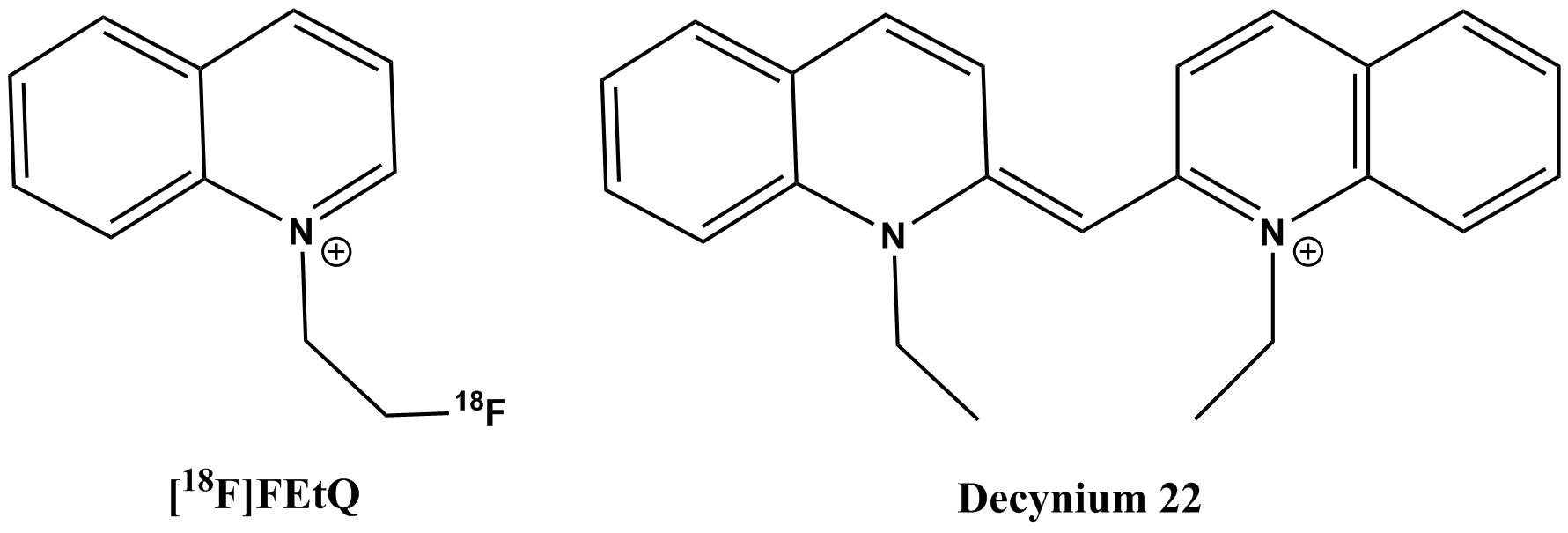

Supplement: Supplementary file 4 — (PNG 39 KB) [file 11307_2022_1728_Fig8_ESM.png]

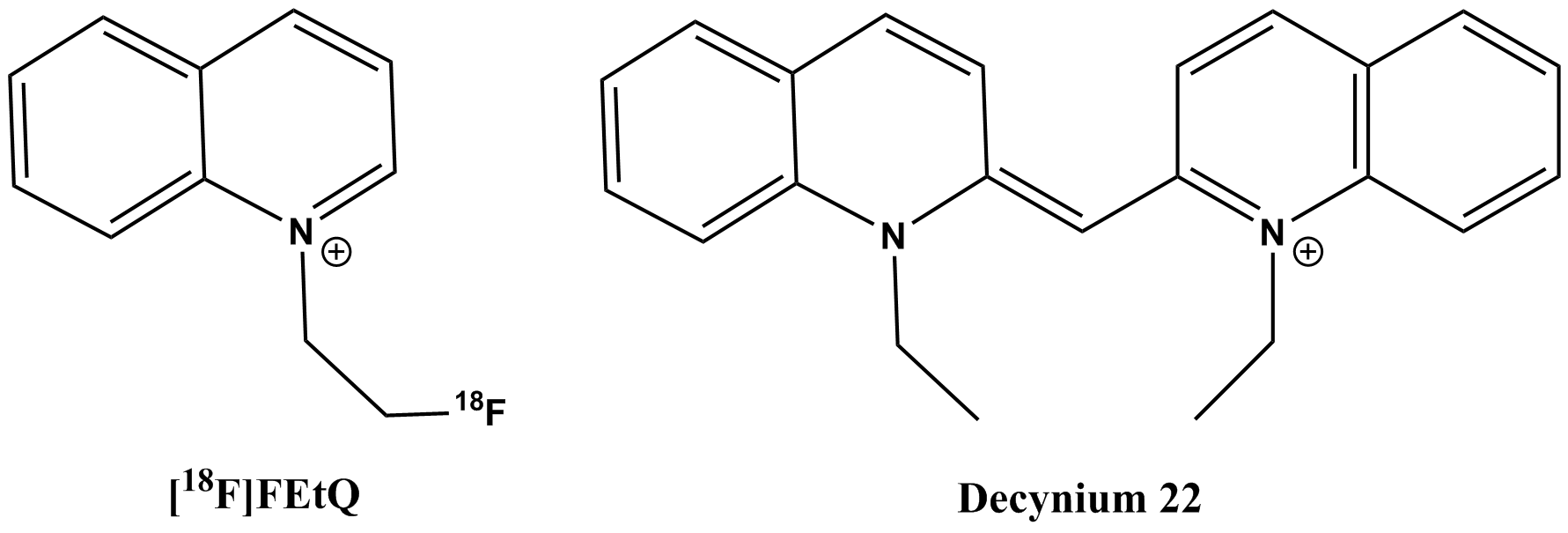

Supplement: Supplementary file 5 — Fig. S2 Chemical structure of [18F]FEtQ and D 22 (TIF 103 KB) [file 11307_2022_1728_MOESM3_ESM.tif]
